# Influence of Different Plant Species on Methane Emissions from Soil in a Restored Swiss Wetland

**DOI:** 10.1371/journal.pone.0089588

**Published:** 2014-02-21

**Authors:** Gurbir S. Bhullar, Peter J. Edwards, Harry Olde Venterink

**Affiliations:** 1 Institute of Integrative Biology, Plant Ecology, ETH Zurich, Zurich, Switzerland; 2 Research Institute of Organic Agriculture (FiBL), Frick, Switzerland; 3 Plant Biology and Nature Management, Vrije Universiteit Brussel, Brussels, Belgium; Beijing Forestry University, China

## Abstract

Plants are a major factor influencing methane emissions from wetlands, along with environmental parameters such as water table, temperature, pH, nutrients and soil carbon substrate. We conducted a field experiment to study how different plant species influence methane emissions from a wetland in Switzerland. The top 0.5 m of soil at this site had been removed five years earlier, leaving a substrate with very low methanogenic activity. We found a sixfold difference among plant species in their effect on methane emission rates: *Molinia caerulea* and *Lysimachia vulgaris* caused low emission rates, whereas *Senecio paludosus*, *Carex flava*, *Juncus effusus* and *Typha latifolia* caused relatively high rates. *Centaurea jacea, Iris sibirica*, and *Carex davalliana* caused intermediate rates. However, we found no effect of either plant biomass or plant functional groups – based on life form or productivity of the habitat – upon methane emission. Emissions were much lower than those usually reported in temperate wetlands, which we attribute to reduced concentrations of labile carbon following topsoil removal. Thus, unlike most wetland sites, methane production in this site was probably fuelled chiefly by root exudation from living plants and from root decay. We conclude that in most wetlands, where concentrations of labile carbon are much higher, these sources account for only a small proportion of the methane emitted. Our study confirms that plant species composition does influence methane emission from wetlands, and should be considered when developing measures to mitigate the greenhouse gas emissions.

## Introduction

Wetlands are the largest natural source of the important greenhouse gas methane (CH_4_), contributing about one-third (80–110Tg yr^−1^) to global emissions [1]–[3]. This methane is produced under anoxic conditions by methanogenic microbes (Archaea) [4]. However, the amounts actually emitted from a wetland soil can be significantly influenced by vascular plants [5]–[8], through their effects upon the production, transport and consumption of methane in soils [4], [9], [10]. These processes vary greatly among plant species, and their net impact upon methane emissions can range from negative to positive [5], [10]–[12]. Much of our understanding about these processes comes from mesocosm experiments or studies conducted in environments lacking substrate uniformity. To better understand why plant species influence methane emissions differently, comparative studies are needed in wetland ecosystems under homogeneous field conditions.

In an earlier study, we showed that graminoids tended to transport more methane from rhizosphere to atmosphere than forbs [13], [14], and other workers have also found plant growth form or functional type to be a factor influencing methane emissions from wetlands [15]–[17]. However, most of this information has come from mesocosm experiments, and field studies and needs to confirm whether the observed differences are ecologically important. Furthermore, it would be useful to have more quantitative information on this topic as a basis for modelling studies and for designing mitigation strategies [18], [19].

Methane emissions from wetlands may also be influenced, either directly or indirectly, by a range of environmental factors such as water table, temperature, pH, nutrients and soil carbon [20]–[23]. For example, by affecting rates of root exudation and rhizosphere oxidation, factors influencing plant productivity could have an indirect influence upon methane emissions. Indeed, some studies have found a positive relationship of emissions with plant productivity [24], [25], while others have reported either no relationship or a negative one [26], [27]. In our mesocosm experiments, we found that plant species from less productive habitats caused higher rates of methane emission than species from more fertile habitats [10], [28]. This was apparently due to higher rates of organic acid exudation by species from less productive habitats, which increased the carbon available to methanogenic bacteria [10], [14].

Our aim in this study was to understand how plant species and/or functional plant groups (based on growth form and productivity indication values) influence methane emissions under field conditions. For this purpose, we needed a site that was as uniform as possible, to avoid possible species effects being confounded by effects due to heterogeneity in soil and hydrological conditions [29]. We therefore selected a wetland site in northern Switzerland where the top 0.5 m of soil had been removed five years previously as part of a restoration scheme, and the area sown with mixtures of native wetland plants. The species sown included both forbs and graminoids, and plants characteristic of habitats of high and low productivity. We assumed that the restoration treatment had reduced soil heterogeneity, so that emissions would be influenced more by the plant studied than by local site differences. The hypotheses were:

- Plant species differ in their effect on methane emission from wetlands.
- Graminoids cause higher methane emissions than forbs.
- Species from low productive habitats cause higher emissions than those from high productive habitats.
- Methane emissions are negatively related to plant biomass.

## Materials and Methods

The experiment was conducted in a wetland (nature reserve Hütwilersee) in Eastern Switzerland (47°36′42.31” N and 8°50′15.35”E) during May 2010. The permission for the conduct of research at this site was granted by Stiftung Seebachtal, CH-Frauenfeld, the foundation owning the land and being set in-charge for the entire restoration project “Seebachtal” by the authorities of canton Thurgau. The vegetation of the study site is composed of both annual and perennial forbs and graminoids. For much of the year, the water table at the study site is at or close to the soil surface, though it may drop to 60 cm in dry periods during the summer. The site is mown once a year in autumn, and the cut material is removed. This management is similar to that practised over the centuries when the site was used as a traditional wet meadow. Preliminary tests conducted in the laboratory showed very low methanogenic activity in soil samples from the site, which increased substantially when glucose was added as a carbon source. This suggests that the unaltered substrate had very low concentrations of labile carbon, presumably as an effect of removing the top 0.5 m of soil five years previously.

The plant species studied were five forbs (*Centaurea jacea* L., *Iris sibirica* L., *Lysimachia vulgaris* L., *Senecio paludosus* L., *Typha latifolia* L.) and four graminoids (*Carex davalliana* SM., *Carex flava* L., *Molinia caerulia* (L.) Moench, *Juncus effuses* L.) (Table 1). Because the area was densely vegetated, there was no opportunity to make reference measurements of methane emission from bare soil. Also, no comparable site without topsoil removal was available for sampling.

**Table 1:** Plant species studied in the experiment ranked according to their habitat preference based on Ellenberg & Move fertility indication values.

Species	Species code	Plant type	Ellenberg N-value*	Move N-value**
*Molinia caerulea*	MC	Graminoid (grass)	1	3.15±1.11
*Carex davalliana*	CD	Graminoid (Sedge)	2	-
*Iris sibirica*	IS	Forb (Monocot)	2	-
*Carex flava*	CF	Graminoid (Sedge)	2	-
*Centaurea jacea*	CJ	Forb (Dicot)	−99	4.76±1.01
*Juncus effuses*	JE	Graminoid (Rush)	4	4.79±1.13
*Lysimachia vulgaris*	LV	Forb (Dicot)	−99	4.88±1.17
*Typha latifolia*	TL	Forb (Monocot)	8	5.95±0.90
*Senecio paludosus*	SP	Forb (Dicot)	6	6.09±0.78

*[42]**[43].

The methane measurements were made during the last week of May 2010, using a Photo Acoustic Field Gas-Monitor type 1412 (Innova AirTech Instruments) fitted with a moisture filter [13]. The water table was recorded from the measurement tubes already installed for another experiment running in the same field. It had rained almost daily for the preceding three weeks, and the water table when the measurements were made was close to the soil surface level throughout the site. For each of the nine species, we selected six replicate spots where the plant was growing well. All spots were spread randomly within an area of 0.6 ha. At each of these spots, one or a few individuals (depending upon species) were carefully selected and covered with a transparent Plexiglas chamber (diameter 19.28 cm, height 60.17 cm), which was placed over the aboveground part of the plant(s) and was pushed about a centimetre into the moist soil for making it air tight. The change in methane concentration inside the chamber during a period of three hours was recorded, and methane emission was expressed per square meter (1 m^−2^) of soil surface (calculated from the diameter of the chamber).

Measurements were always made between 10:00 and 14:00 hours. Weather conditions varied from rainy to partly cloudy, and the ambient air temperature ranged between 11 and 20°C. Methane density values corresponding to air temperature were used for calculating absolute methane emissions (measured in μg m^−2^ hr^−1^). We did not extrapolate these values over longer periods because methane emissions from wetland soils are known to exhibit strong diurnal and seasonal variation [7], [30]–[32]. After each measurement, the total aboveground plant biomass from the measured spot was harvested, and a soil sample was collected from a depth of 0–30 cm. The harvested material was dried at 70°c for 48 hours and weighed to determine aboveground biomass. Soil moisture was determined by drying a portion of the sampled soil at 105°C for 24 hours. Soil pH was determined in distilled water. Nitrate and ammonium concentrations extracted with 1M KCL were determined colorimetrically using a (FIASTAR 5000) flow injection analyser [33]. During the measurement period, soil was almost always saturated with water table between 6 and 11 cm below soil surface and soil moisture (0–30 cm depth) between 86 and 89 percent. Soil pH at all sampling points was almost uniform at close to 6. Soil extractable nitrate was below detection limits, and ammonium was between 8 to 17 mg N kg^−1^ dry soil.

Differences among plant species in methane emissions and environmental parameters (temperature, pH, nitrogen, soil moisture) were tested by means of ANOVA and Tukey test. To confirm with the assumption of homogeneity of variance and normality, the data were log-transformed prior to data analysis. We also calculated linear regression between methane emission rates and plant biomass for each species. The data were analysed using statistical software R, version 2.8.1 [34].

## Results

**Figure 1:**
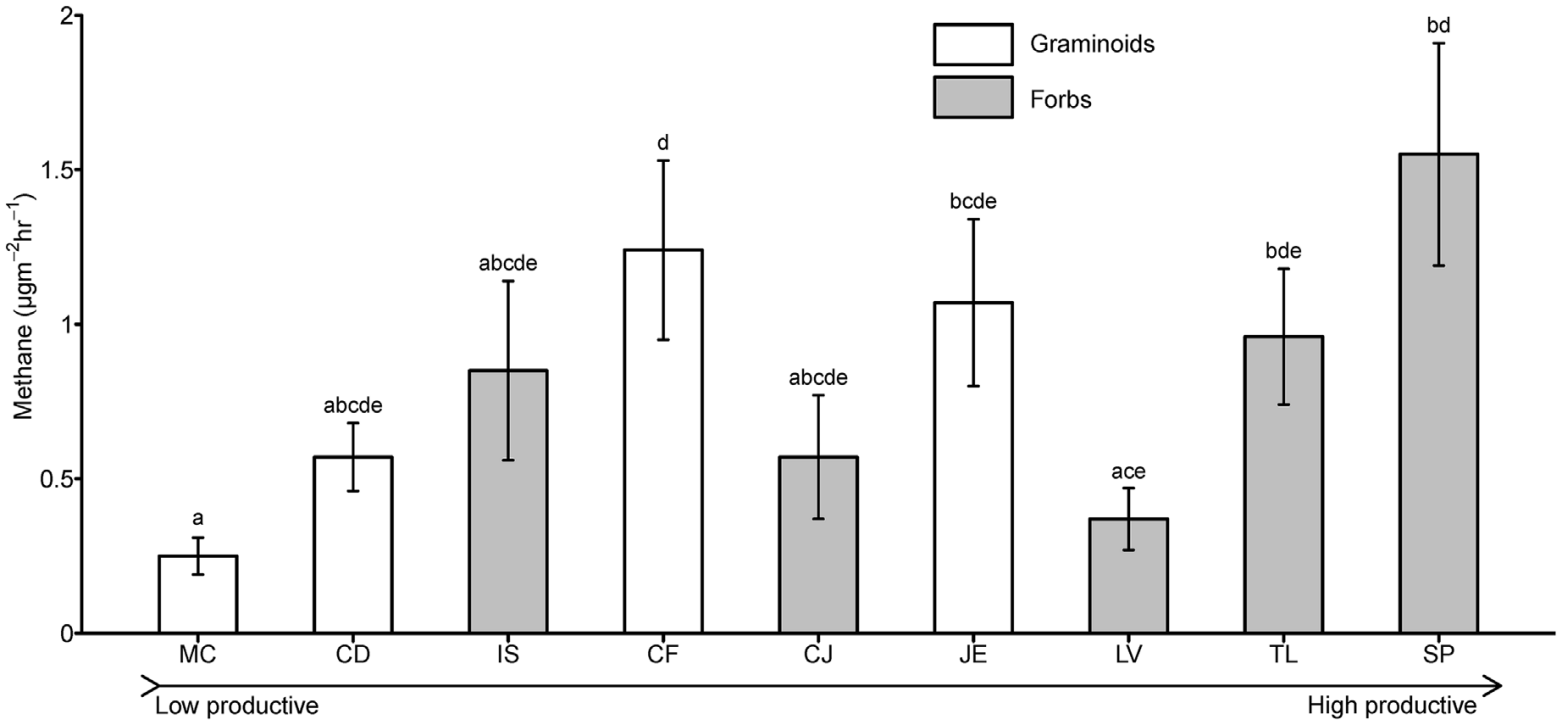
Mean CH_4_ emission rates from different plant species, arranged in order of increasing fertility indication [42], [43] from left to right on x-axis (see Table 1). MC: *Molinia caerulia* (L.) Moench, CD: *Carex davalliana* SM., IS: *Iris sibirica* L., CF: *Carex flava* L., CJ: *Centaurea jacea* L., JE: *Juncus effuses* L., LV: *Lysimachia vulgaris* L., TL: *Typha latifolia* L., SP: *Senecio paludosus* L. Error bars represent original data whereas statistics are based on log transformed data.

Methane emissions varied significantly among plant species (Fig. 1). *Molinia caerulea* was associated with the lowest emissions, while *Senecio paludosus* and *Carex flava* had the highest emissions. Emissions from *Juncus effusus* and *Typha latifolia* also tended to be higher but were statistically not different from other species. Methane emissions were sensitive to changes in air temperature, but this did not affect the comparison among different plant species since the replicates within species were spread over different days and temperatures.

We found neither significant difference between graminoids and forbs (*p* = 0.95), nor between species with low and high Ellenberg N-values (below and above 4; *p* = 0.49). Methane emissions were also not significantly related to aboveground plant biomass; and emissions from different species were not significantly related to any of the measured site factors (Table 2).

**Table 2:** Results of linear regression between methane emission and environmental factors.

	Based on all data points (log-transformed)		Based on mean value per species	
Factor	R squared	p-value	R squared	p-value
Plant Biomass	0.005	0.61	0.011	0.78
Soil pH	0.004	0.65	0.049	0.56
Soil extractable NH_4_– Nitrogen	0.009	0.49	0.120	0.35
Air Temperature	0.088 *	0.03	0.001	0.92
Soil moisture	0.001	0.86	0.112	0.37
Water table	0.012	0.42	0.075	0.47

Soil variables were measured in the top 30 cm.

P≤0.05; **P≤0.01; ***P≤0.001.

## Discussion

Methane emissions from natural wetlands have been reported to range between 0 and 660 mg m^−2^ day^−1^ [1], [2], [30], [35]. At our site, they varied from 0.25 to 1.55 μg m^−2^ hr^−1^, which is lower by a magnitude of thousands than those measured at many other temperate wetlands [27], [36]. Moore and Knowles [37] have reported similarly low rates of emission from an ombrotrophic bog in Quebec (Canada), where they were perhaps caused by very slow decomposition of *Sphagnum* litter and suboptimal pH for methanogenesis. At our site, the low methanogenic activity was probably a consequence of removing the topsoil five years previously, thereby reducing concentrations of labile carbon in the substrate. Indeed, in a separate mesocosm study, we found that methane emissions increased dramatically when glucose was added to the substrate obtained from the same field site. In addition, the site was mown each year and the hay removed, so that little aboveground biomass entered the soil as litter. Thus, unlike most wetland sites, methane production in this site was probably fuelled chiefly by root exudation from living plants and from root decay [38]. The very low overall methane emission, which is one of the main results from our study, underlines an important aspect of wetland management and methane mitigation. It suggests that topsoil removal coupled with regular biomass removal can reduce methane emissions for an extended period – although we can only base this conclusion on comparison with other sites since control sites without topsoil removal were not available in our site. Furthermore, it must be noted that we measured methane emission on only one occasion, chosen for the hydrological homogeneity caused by several weeks of continuous rain.

Despite the low emissions, the site was well suited for comparing methane emissions associated with different plant species. In support of our first hypothesis, methane emission from soil at various points varied significantly according to the plant species growing there. Previous studies have also shown that plant species differ in their influence on methane emissions, though these were conducted either in mesocosms [10], [13], [17], [22] or under rather heterogeneous field conditions [8], [11] where microtopographic or other factors might have affected the results [5], [36]. In our field site, we found no correlation between environmental parameters and methane emissions from different plant species, presumably because of the rather uniform site conditions [20], [23].

In an earlier greenhouse experiment comparing a large set of wetland plants, we found that graminoids had a greater capacity to transport methane from soil to atmosphere (chimney effect) than forbs [13]. This effect appeared to be largely a result of differences between the root systems in the two functional groups [39], [40]. No such relationship was evident in the current study, however, presumably because too little methane was produced to detect any differences in transport capacity.

Results of this field study do not support our third and fourth hypothesis, which were based on mesocosm experiments showing higher emissions caused by species of less productive habitats and decreasing emissions with increase in plant productivity [10], [26]–[28]. Higher methane emission from mesocosms with species adapted to less productive habitats was attributed to higher carbon exudation rates in these species [10]. Because our study site was depleted of labile carbon, we had expected to find similar effects in the field, arguing that under these conditions differences in exudation rates among species would be detectable as differences in methane emissions. But we found neither a difference among plant species from low and high productive habitats nor a significant relationship between methane emissions and plant biomass (Table 2). Some field studies have shown a positive correlation between methane emissions and vegetation biomass or productivity [12], [24], [25], while mesocosm experiments have shown the opposite effect [10], [27], [28]. This difference may have arisen because the amount of methane actually emitted is the resultant of several processes, but which of these dominates may vary according to the conditions. For example, under conditions at our site, the addition of carbon through root exudation was probably the primary controlling mechanism, which in turn depended upon current photosynthetic activity of the vegetation [24], [41]. In contrast, when plants are grown under controlled conditions such as in mesocosm experiments, the rhizosphere oxidation by the plant roots might be of relatively greater significance [26], and therefore plants with larger biomass are able to exert a relatively larger influence through radial oxygen loss by occupying all the rhizosphere space available in the mesocosm. Thus, mesocosm experiments do not necessarily reflect processes operating in the field.

In conclusion, this study demonstrates that plant species vary in their capacity to influence methane emissions even under conditions where methanogenesis is limited by a shortage of labile carbon. However, the very low fluxes measured in our study suggest that only a small proportion of the methane emitted from most wetlands can be due to root exudation. This in turn suggests that plants have a much greater potential to influence emissions by transporting methane internally or by altering redox conditions in the soil. Finally, our results indicate that management practices such as removing topsoil and mowing the vegetation could be an effective way of reducing methane emissions from wetlands. Further research would be needed, however, to determine whether any benefits would exceed the ‘carbon debt’ associated with removing the top soil.
